# Development of multi-criteria decision analysis (MCDA) framework for off-patent pharmaceuticals – an application on improving tender decision making in Indonesia

**DOI:** 10.1186/s12913-018-3805-3

**Published:** 2018-12-29

**Authors:** Andras Inotai, Diana Brixner, Nikos Maniadakis, Iwan Dwiprahasto, Erna Kristin, Agus Prabowo, Alfi Yasmina, Sigit Priohutomo, Bertalan Németh, Kalman Wijaya, Zoltan Kalo

**Affiliations:** 1 Syreon Research Institute, Mexikói str. 65/A, Budapest, 1142 Hungary; 2 Department of Health Policy and Health Economics, Eötvös Loránd University (ELTE), Pázmány Péter sétány 1/A, Budapest, 1117 Hungary; 3 Department of Pharmacotherapy, University of Utah College Of Pharmacy, Pharmacotherapy Outcomes Research Center, Salt Lake City, UT USA; 4 Health Services Organization and Management, National School of Public Health, Athens, Greece; 5 Department Of Pharmacology & Therapy, Faculty Of Medicine, Universitas Gadjah Mada, Yogyakarta, Indonesia; 6 National Public Procurement Agency of the Republic of Indonesia, Jakarta, Indonesia; 7 Department Of Pharmacology, Faculty Of Medicine, Lambung Mangkurat University, Banjarmasin, Indonesia; 8 Coordinating Ministry for Human Development and Culture Building, Jakarta, Indonesia; 9 Abbott Established Pharmaceutical Division, Basel, Switzerland

**Keywords:** Off-patent pharmaceuticals, Multi-criteria decision analysis, Tender, Bioequivalence, Developing countries

## Abstract

**Background:**

Off-patent pharmaceuticals (OPPs) hold vital importance in meeting public health objectives, especially in developing countries where resources are limited. OPPs are comprised of off-patent originals, branded generics and unbranded generics; nonetheless, these products are not identical and often there are differences in their equivalence, manufacturing quality standards and reliability of supply. This necessitates reconsideration of the lowest price policy objective in pharmaceutical decision making. The aim of this study was to develop a Multi-Criteria Decision Analysis (MCDA) framework through a pilot workshop to inform the national procurement of OPPs in Indonesia.

**Methods:**

An initial list of potentially relevant criteria was identified based on previous work and a literature review. In a 2-day pilot policy workshop, twenty local experts representing different stakeholder groups and decision-making bodies selected the final criteria, approved the scoring function for each criterion, and assigned weights to each criterion.

**Results:**

An MCDA framework was proposed for OPP drug decision making in developing countries, which included price and 8 non-price criteria. Based on the pilot policy workshop 6 + 1 criteria were considered relevant for Indonesia: pharmaceutical price (40% weight), manufacturing quality (18.8%), equivalence with the reference product (12.2%), product stability and drug formulation (12.2%), reliability of drug supply (8.4%), real world clinical or economic outcomes, such as adherence or non-drug costs (4.2%) and pharmacovigilance (3.6%).

**Conclusions:**

According to the pilot policy workshop, other criteria apart from price need to be strengthened in the tendering process. The introduction of additional criteria for OPP procurement in an MCDA framework creates incentives for manufacturers to invest into improved manufacturing standards, equivalence proof, product quality, reliability of supply or even additional real-world data collection, which ultimately may result in more health gain for the society.

## Background

### Current policy objectives for OPPs

Off-patent pharmaceuticals (OPPs) are among the most widely used health care technologies in many diseases with a large impact on public health. As first line treatments for many chronic conditions [1–3], the success of public health policies in improving the health status of total population highly depends on the success of OPP policies, especially in developing countries where health care resources are even more limited to cover significant unmet need [4]. The lowest price is often considered the main efficiency criterion of OPP policies by health care decision makers. While this approach assumes that OPPs (comprising off-patent originals, branded generics and unbranded generics) are associated with the same attributes as their reference products in terms of quality, real world effectiveness or stability, there are shortcomings even in developed countries with high regulation standards [5]. Obviously, even more concerns could be raised against this policy objective in developing countries with a less highly regulated pharmaceutical system.

### Limitations of lowest price policy objectives in developing countries

In the developed countries, bioequivalence (BE) has been fully adapted as a requirement for generics. However, in most developing countries even pharmaceutical equivalence (PE) has not yet been implemented for copies of original OPPs. Similarly, while in developed countries, Good Manufacturing Practice (GMP) according to EU/ Pharmaceutical Inspection Co-operation Scheme (PIC/S) standards including regular audits is considered to be a guarantee of constantly high product quality, in several developing countries even the lower WHO GMP standard has not yet been adapted [5]. Capacity problems of manufacturers, lack of profitability, inefficiencies of regulatory processes or pharmaceutical tendering, increasing demand, quality issues, unstable political or economic environments and inefficient distribution systems are among the concerns which are even more specific to increasing drug shortages not only in developing countries but also in developed European countries [6, 7]. These factors necessitate the consideration of multiple criteria in drug decision making especially in developing countries.

### Current procurement framework for off-patent pharmaceuticals in Indonesia

Indonesia has been using the Universal Health Coverage (Jaminan Kesehatan Nasional, JKN) since 2014, wherein the e-Purchasing procedure has been established for drug procurement for the national formulary. The drug prices and volumes to be annually utilized for the JKN program are listed in the E-Catalogue system by the National Commitment Officer (Pejabat Pembuat Komitmen, PPK). This procurement data is included in the National Procurement Portal (later known as Drug Procurement List) which is classified into single and multiple source (OPP) products. Single source products are evaluated by a committee through negotiation processes and multiple source products through public tenders. Tender evaluations are determined by price and non-price criteria (for example, quality and supply capacity) [8, 9]. However, the limited consideration of critical parameters in the current methodology, including failures in hospital estimations on the required drugs calculation (RKO), E-Catalogue and manual procurement as well as variation of drug distribution processes all lead to the nationwide pharmaceutical supply shortages for several drugs [10, 11].

### Need for multi-criteria decision making in OPPs

A Multi-Criteria Decision Analysis (MCDA) framework could support the decision-making process by aggregating multiple policy objectives [12]. It is a methodology for appraising alternatives on individual, often conflicting criteria, and combining them into one overall appraisal [13, 14]. Each criterion considered in MCDA has a relative weight within the final evaluation reflecting its relative importance within the decision context. The subject of the evaluation is scored according to how it performs within each criterion. The aggregation of the scores received for each criterion multiplied by their relative weights results in a composite score. The scores of each alternative option can be compared to improve the objectivity, transparency and consistency of decision making. This tool has been increasingly used in health care to facilitate a wide spectrum of decision problems [15, 16]. For example, application of MCDA in the off-patent sector is now considered in China to inform Beijing tendering decisions and Egypt for vaccine tenders [17, 18].

### Previous work in this concept

In order to improve the evidence base of theoretical and practical implementation levels of making decisions in the off-patent sector in developing countries, an International Outcomes Research Board (IORB) including experts from academic, public and private sectors was established and held multiple workshops. One of the key deliverables of these workshops was the MCDA ‘Simple Scoring’ tool adapted for off-patent products to overcome one frequently mentioned barrier of MCDA, which is, that it is too complex for local decision makers to use, especially without previous experience. The Board identified a list of 22 criteria that could potentially be used to evaluate and make multiple policy decisions about off-patent medicines including their pricing, reimbursement, formulary listing, drug procurement, prescription, authorization and research. The 22 criteria, presented on September 6th, 2016 in the ISPOR MCDA Simple Scoring Educational Forum workshop in Singapore, represents a solid and scientifically rigorous basis for off-patent product evaluations which also allows for customization to the needs of developing countries [5].

The aim of this study was to develop, test and fine-tune a multi-criteria decision analysis framework on the basis of the MCDA ‘Simple Scoring’, designed to facilitate the decision-making process during the national procurement of off-patent pharmaceuticals in Indonesia.

## Methods

The development of the MCDA framework consisted of a preparatory part with a literature review and multiple teleconferences with Indonesian academic experts, and a 2-day on-site workshop in Jakarta with 20 experts representing different stakeholders in the national procurement of OPP procurement.

### Pre-workshop preparation

Seven potential decision contexts were identified in the literature review for MCDA application of OPPs including market authorisation, pricing, coverage/reimbursement, national or hospital formulary listing and national procurement or hospital tenders [5]. The Indonesian national procurement of OPPs was considered to be a relevant decision problem to be improved with the use of MCDA methodology.

A five-step evidence-based approach was used to prepare the initial criteria list. The 22 items in the ‘Simple Scoring’ MCDA framework by Brixner et al. was the starting point for criteria selection [5]. The second step was to extend the scope of the ‘Simple Scoring’ framework with an additional literature review. The literature review identified 7 additional criteria. As a third step, in multiple teleconferences local academic experts from Universitas Gadjah Mada (UGM) provided an overview on current national procurement processes in Indonesia and indicated potential concerns with the current provision of OPPs. The objective of the fourth step was to reduce the criteria list to below 10–11 items in accordance with the recommendations of the International Society for Pharmacoeconomics and Outcomes Research (ISPOR), to reduce the complexity, but not sophistication, of it for future use [12]. Based on local insights the 29-item criteria list was reduced by excluding several criteria 1) with limited relevance for national procurement of OPPs in Indonesia (e.g. budget impact, cost effectiveness, order of entry) and 2) without explicitly defined scoring functions (MCDA scores should be derived by clearly defined rules or functions aiming to convert performance measurements into scores). In addition, to meet the principles of completeness, non-redundancy, non-overlap and preference independence recommended by ISPOR [12], some of the remaining criteria were merged. For example, Pharmaceutical equivalence, Interchangeability and Bioequivalence were all merged into a new criterion called ‘Equivalence with the reference product’; and Technical assistance, Disease awareness & education and Continued Medical Education were merged into ‘Added value services related to the product’. In the end, the draft list of the MCDA framework contained 8 + 1 mutually exclusive criteria, including 1) Equivalence with the reference (original) product, 2) Real world clinical or economic outcomes such as adherence or non-drug costs, 3) Product stability and drug formulation, 4) Quality assurance, 5) Macroeconomic benefit, 6) Reliability of drug supply, 7) Pharmacovigilance, 8) Added value service related to the product and 9) Price. The initial criteria list, along with their intended definitions and corresponding performance categories are listed in Table 1. As the final step, the relevance of this initial criteria list was validated by local academic experts from UGM.

**Table 1:** Draft list of criteria and performance categories

Criteria	Intended definition	Performance categories	Test case 1	Test case 2	Test case 3	Test case 4	Pilot workshop outcome of criteria
Equivalence with the reference (original) product	to capture evidence on health outcomes from pharmaceutical-, bioequivalence- and clinical trials (efficacy data from controlled clinical settings)	- No data on pharmaceutical equivalence					Included
		- Pharmaceutical equivalence					
		- Interchangeability defined based on local criteria	x				
		- Bioequivalence proven based on local criteria		x	x		
		- Bioequivalence proven based on European EMA or US FDA criteria				x	
		- Therapeutic equivalence proven in clinical trial					
		- Improvement in efficacy and/or safety based on clinical trial data					
Real world clinical or economic outcomes such as adherence or non-drug costs	to capture evidence on health outcomes (effectiveness) and costs from real-world data	- No real world data on equal a) tolerability, b) adherence and persistence, c) non-drug cost	x				Included
		- International real world data on either equal a) tolerability, b) adherence and persistence, c) non-drug cost		x			
		- Local real world data on either equal a) tolerability, b) adherence and persistence, c) non-drug cost			x	x	
		- International real world data on improvement in a) tolerability, b) adherence and persistence, c) non-drug cost					
		- Local real world data on improvement in a) tolerability, b) adherence and persistence, c) non-drug cost					
Product stability and drug formulation	to capture evidence on stability and drug formulation	- No data on product expiry or stability					Included
		- Data on non-inferior product expiry or stability in local environment	x				
		- Data on improved product expiry			x	x	
		- Data on improved product stability in local environment		x			
		- Data on improved product expiry and stability in local environment					
Quality assurance	to capture evidence on manufacturing- and product quality-, and standardisation	- Limited information on quality assurance					Included
		- Local/non GMP quality assurance only for active product ingredient	x				
		- Local/non GMP quality assurance for the entire manufacturing process		x	x		
		- WHO GMP certification				x	
		- EU or PIC/s GMP					
Macroeconomic benefit	to capture wider economic benefits of selecting the medicine (e.g. tax, investment, employment etc.)	- The manufacturer has no local investment in the country					Excluded by majority voting
		- The manufacturer has minor local investment in the country	x	x			
		- The manufacturer has moderate local investment in the country			x		
		- The manufacturer has significant local investment in the country				x	
Reliability of drug supply	to capture the stability and reliability of drug supply (history and future gurantee)	- Major and multiple problems in the last 5 yrs					Included
		- Minor and fairly frequent problems in the last 5 yrs	x				
		- Single precedence of supply problems in the last 5 yrs		x			
		- No precedence of supply problems in the last 5 yrs			x	x	
		- Manufacturer is financially capable and willing to guarantee supply					
Pharmacovigilance	to capture data collection and assessment on adverse events of pharmaceuticals	- No pharmacovigilance system	x				Included
		- Qualified person for pharmacovigilance		x			
		- Qualified person and sophisticated system to collect pharmacovigilance data			x	x	
Added value service related to the product	to capture extra services provided alongside the drug with quantifiable and demonstrated outcomes/benefits	- No program or service	x				Excluded by majority voting
		- Availability of value added service		x			
		- Major value added service with demonstrated outcomes			x	x	
Price	Acquisition cost of the pharmaceutical product compared to the lowest price available	N/A	2200 IDR	2900 IDR	3000 IDR	3800 IDR	Included

IDR - Indonesian Rupiah

### Methodology of ranking and weighting criteria

The selection of ranking and weighting methodology was based on the typology of the ISPOR MCDA Task Force [12] and the review of scientific literature on MCDA methodology. In an initial screening, the following ranking and weighting methods were excluded: 1) those with less of a solid theoretical foundation, 2) those which were not able to be implemented through a voting system and 3) those methods which were time consuming considering the duration of the 2-day on-site workshop. Those methods remaining on the short list after the initial feasibility screening were ranked according to their simplicity/implementation time and solidity of methodology. Finally, the modified Simple Multi-Attribute Rating Technique (SMART) method for ranking and swing weighting were selected based on the decision of an expert panel as an optimal balance between feasibility and scientific rigor [19, 20].

### Preparation of an MCDA excel framework

An MCDA tool was developed in MS-Excel, with spreadsheets containing all criteria, the corresponding performance categories for each criterion and their scoring functions. Criteria weights were to be entered based on the SMART and swing weighting voting results of the workshop. The performance matrix (main evaluation sheet) of the Excel tool was designed to be applicable for routine use when comparing multiple competing products in the national procurement process. The performance of each product according to different criteria can be selected from drop-down menus linked to the corresponding performance categories. The draft MCDA tool was designed to translate the performance matrix of each product to an aggregate MCDA score according to the scoring function and weight of each criterion, and indicate the ranking among competing alternative medicines.

### Initial validation by key local experts

The proposed methodology on ranking and weighting, the draft criteria list, initial performance categories and proposed scoring functions were all discussed with-, and validated and approved by academic experts from different universities in Indonesia during two teleconferences. These experts were also invited to the policy workshop. To facilitate knowledge transfer even before the workshop for those with more limited background information on MCDA, a customised pre-reading material was developed and circulated among workshop participants.

Additionally, a set of four competing test cases was developed and pre-calibrated to simulate real world tendering decisions. Each test case represented a full drug profile described in a one-page summary report, containing information on how the pharmaceutical product performed according to each criterion.

### Outline of the workshop

A 2-day on-site workshop was organized with the participation of key local stakeholders involved in the public procurement of off-patent pharmaceuticals. An overview of the policy landscape for procurement of OPPs was provided along with a background on the criteria for consideration in procurement decisions. The key objective of this workshop was to 1) select and approve the relevance of criteria in the Indonesian procurement process of OPPs 2) rank and weight the criteria 3) approve scoring functions of each criterion as well as 4) validate and fine-tune the MCDA framework based on reference cases.

### Weight and scoring function of pharmaceutical price

According to a recently published guidance on the implementation of MCDA framework in developing countries [21], MCDA development should be started from the current decision-making criteria. Consequently, the first step was to determine the weight of pharmaceutical acquisition cost in the new MCDA framework. Workshop participants had to choose between values from 5 to 100% by 5% increments through anonymous voting implemented via a voting machine. The median value was calculated to reduce the impact of outliers. The second step was to determine the scoring function for acquisition cost (price). Participants were asked to vote on how much should be the excess price, above which they reward a product with 0% score for the Price criterion. This excess price was determined compared to the lowest-price option rewarded with maximum points for Price criterion. In this step, a + 200% excess price means that products with prices at least 200% higher (i.e. three times or more expensive) than the lowest price candidate will receive 0% of the scores according to the Price criterion (cut-off point), while products with a 100% higher price (i.e. twice as expensive) will receive 50% of the maximum scores that can be granted based on the Price criterion. The full 100% of price scores are to be awarded to the lowest price option. Therefore, the price function was linear and decreasing from the lowest price product (with 100% of the price score assigned) to the cut-off point (with 0% of the price score assigned). Participants could select the excess price (i.e. the cut-off point) between + 25% to + 500% to modify the steepness of a scoring function for prices and the median values were calculated to determine the cut-off point. Having a price scoring function independent of the price distribution of the competing products (i.e. by not linking the zero point for price to the most expensive product) prevents differences from being too large within the points awarded when product prices are in a small range or being too small in points awarded when the product prices are in a large interval. In other words, the steepness of the scoring function can be adjusted to the expected price distribution of the competing products.

### Creating scoring functions and ranking of non-price criteria

As an introduction to the third step, participants were presented the 8-item list of non-price criteria, including 1) the definition of criteria, 2) the performance categories (see Table 1) and 3) the proposed scoring function for each criterion. The draft scoring function for each non-price criterion was defined by percentages of scores assigned to the corresponding ordinal-scale performance categories and was validated by local experts before the workshop. A higher percentage indicated better performance, with 100% being the full score that could be awarded any particular criterion, while 0% represented the worst score for performance of any criterion. Very poor performance in some key criteria resulted in their exclusion from the assessment (exclusion category). At this stage participants had the opportunity to add or delete items from the criteria list and the performance categories as well as to adjust the scoring function by majority voting. Once attributes of non-price criteria were discussed and agreed upon, as the third exercise participants were asked to rank the criteria based on their importance according to the modified SMART method. As a warming-up exercise, participants were presented a ‘worst case scenario’ (i.e. a hypothetical product performing in all the criteria at their worst levels). Participants were then asked to identify and vote on that criterion which they thought should be moved from the worst level to the best performance level to improve the performance of the hypothetical product. This was done to identify the most important criterion. Then participants were asked to vote on another criterion to be moved from the worst level to the best performance level again, to improve the performance of the hypothetical product. This identified the second most important criterion etc. In the end, the modified SMART process resulted in a list of the different criteria in order of their importance.

### Weighting non-price criteria

Once the ranking between criteria was established, a swing weighting method was used to estimate the relative weights of the non-price criteria as the fourth step. In the swing weighting, a fixed number of points (initially 10), was assigned to the least important criterion. Ten being the lowest point score, participants voted on how much more points the second least important criterion should be assigned to reflect its relative importance. Participants could select percentages ranging from 0% to + 50% by 5% increments and between + 50% to + 100% (i.e. two times more important) by 10% increments respectively, and the median values were considered. If, as an example, the median of the audience’s votes was + 50%, the second least important criterion received 10 + 10*50% = 15 points. In the next vote, participants had to make their judgement on the subsequent criterion in the priority list, considering the points assigned to the second least important criterion (e.g. 15) as a basis (etc.). The voting process was continued until each of the increasingly important criteria received progressively higher points. Scores for swing weighting can be seen in the Result section. In case of equal ranking, a 0% increment was assigned to indicate that a criterion had the same importance as the previous criterion and thus received the same point values. After assigning points to all criteria, first, the non-price criteria scores identified by participants were summarized and normalized to 100%; resulting in the relative weights for each non-price criterion. Once the relative weights of non-price criteria were elicited based on their progressively higher point values, they were normalized once again, yet this time taking the weight of the Price criterion into account as well, to obtain the final weighted values. Participants were shown the final calculated weights for the price and non-price criteria and then had the opportunity to adjust their opinion on any price weight during a second vote. This completed the draft MCDA framework.

### Validating the framework on test cases

Participants were then provided a set of 4 different test cases. These described the performance of 4 hypothetical drugs according to the criteria of the draft MCDA framework. Participants populated the draft MCDA framework with the test cases to prepare the performance matrix. Finally, as a fifth step, participants had the opportunity to adjust the framework with a special focus on the price weight and the scoring function for price (e.g. cut-off point). Preparation of the initial MCDA framework ended at this stage.

## Results

The draft criteria list, their corresponding performance categories and the hypothetical drug profiles are presented in Table 1. Table 2 describes affiliation of the 20 local workshop participants representing different stakeholder groups with a thorough understanding of the national procurement of OPPs. The majority of them had an affiliation at the Ministry of Health, the local drug regulatory agency and the National Formulary Committee.

**Table 2:** Local workshop participants

Institution	Number of experts	%
Badan Pengawas Obat dan Makanan (BPOM) - Local drug regulatory agency	4	20%
Lembaga Kebijakan Pengadaan Barang Jasa Pemerintah (LKPP) - National Public Procurement Agency of the Republic of Indonesia	1	5%
Kementerian Kesehatan Republik Indonesia - Ministry of Health of the Republic of Indonesia	7	35%
Dewan Jaminan Sosial Nasional - Jaminan Kesehatan Nasional (DJSN – JKN) - National Social Security Council	1	5%
Perhimpunan Rumah Sakit Seluruh Indonesia - Hospital association	1	5%
Komite Nasional Penyusunan Formularium Nasional - National Formulary Committee	4	20%
Gabungan Perusahaan Farmasi Indonesia - Pharmaceutial manufacturers association	2	10%
Total	20	100%

The initial median value for both price weight and the cut-off point was 50% (Individual voting results can be seen in Table 3). Except for some slight changes (some performance categories were re-defined as exclusion categories and amendments were suggested in phraseology), the proposed scoring function was approved by the participants with no further requests for additional criteria. Although, after a thorough discussion, two criteria (Macroeconomic benefit and Added value services related to the product) were considered to be less relevant in Indonesia and were excluded from the criteria list by majority voting (Table 1). The ranking of the remaining 6 criteria is shown in Table 4 (Ranking). (Votes for the swing weighting are summarised in Table 3.) Increment swing weights from the least important criterion to the most important ones were + 15%, + 100%, + 50%, + 0% (meaning equal ranking) and + 50%, respectively (See Table 3, for relative scores on swing weighting, see Table 4). After concluding the relative weights of all non-price criteria and normalizing them to 100% (see Table 4, Weight of non-price criteria), the relative weights of non-price criteria were recalculated in order to complete the total 6 + 1 criteria weights with price weight to be exactly 100% (Table 4, Draft weights). Since most participants preferred no adjustment in the price weight and cut-off point at this stage, this was the final step in developing the initial MCDA framework. After entering the data from the test cases described in Table 1 to the Excel framework, participants adjusted the median price weight to 40% and cut-off point to + 100% (Table 4, Final weights of initial MCDA).

**Table 3:** Results of voting

		n	+ 0%	+ 5%	+ 10%	+ 15%	+ 20%	+ 25%	+ 30%	+ 35%	+ 40%	+ 45%	+ 50%	+ 55%	+ 60%	+ 65%	+ 70%	+ 75%	+ 80%	+ 85%	+ 90%	+ 95%	+ 100%	Median
Price weight (initial)		16	1								5		8					1					1	50%
Swing weighting	vote 1	16	4	1	3		1	1				1	3								1		1	15%
	vote 2	15			1			1					2		1								10	100%
	vote 3	16	4		1								4		2		1		1				3	50%
	vote 4	16	16																					0%
	vote 5	15	2		1		1		1				7				1						2	50%
Price weight (adjustment)		15							1		7		7											40%

**Table 4:** Criteria weights

Criterion	SMART Ranking	Swing weighting (see Table 3)	Relative scores for swing weighting	Weight of non-price criteria	Draft Weights	Final weights of initial MCDA
Price advantage	N/A				50.0%	40.0%
Quality assurance	1	Vote 5: + 50%	51.75	31.4%	15.7%	18.8%
Equivalence with the reference (original) product	2	Vote 4: + 0% (=equal importance)	34.5	20.8%	10.4%	12.5%
Product stability and drug formulation	2	Vote 3: + 50%	34.5	20.8%	10.4%	12.5%
Reliability of drug supply	3	Vote 2: + 100%	23	14.0%	7.0%	8.4%
Real world clinical or economic outcomes such as adherence or non-drug costs	4	Vote 1: + 15%	11.5	7.0%	3.5%	4.2%
Pharmacovigilance	5	N/A	10	6.0%	3.0%	3.6%

The objective of the final section of the workshop was to create an action plan on the policy implementation of repeated use MCDA in Indonesian OPP procurement (Table 5). Participants from multiple affiliations confirmed the importance of disseminating the results in a white paper and in a scientific manuscript. Industry representatives highlighted the relevance of transparency to improve the tendering process. This necessitates improving the clarity of requirements when submitting dossiers for off-patent tenders, in part due to manufacturers are not confident in being successful in the tender processes and due to economic reasons because they cannot reduce their prices to the expected price level. Considering multiple winners in each tender may facilitate the willingness of manufacturers to participate in the tender process. Opportunity for revision and adjustment of weights and scoring functions after evaluating the reference cases was considered to be very important in improving the appropriateness of the MCDA tool. As a consequence, gradual implementation of MCDA in policy decisions was recommended. After a 1-year pilot phase, weights and scoring functions should be revised and adjusted according to the initial experience based on routine application of the initial MCDA tool in the pilot period.

**Table 5:** Development of MCDA tool for repeated use

Process		Steps	Deliverable
Development	Desk research	Defining the decision problem	MCDA objectives
		Initial selection and structure of criteria	Draft list of criteria
		Initial scoring functions for criteria	
	Policy workshop	Final selection of criteria	Final criteria list
		Scoring functions for criteria	
		Weighting the criteria	Criteria weighting
Initial dissemination		Initial MCDA tool is released with submission template	Publication (with or without training)
Policy application	Pilot - Repeated use of initial MCDA tool	Manufacturers submit necessary data based on MCDA submission template	Performance matrix
		Secretariat of decision-making body validates information submitted by manufacturers	
		Calculation of aggregate scores	
		Final review of alternatives’ performance by members of decision-making committee	
		Interpretation and reporting	MCDA recommendation
		Policy decision	Final decision
	Revision of MCDA	Revision of initial MCDA tool based on early experiences	Final MCDA tool
		Policy workshop to finalize MCDA tool	
Final dissemination		Final MCDA tool is released with submission template	Publication (with or without training)

## Discussion

### Relevance of test cases in adjusting weight and scoring function of price

The weight of acquisition cost is one of the most crucial criteria in MCDAs use for the procurement of pharmaceuticals. There should be an optimal balance between price weights that are too high suggesting that no meaningful improvement is recommended in the system and weights that are too low suggesting it will receive little support by policy-makers or payers. The settled weight of price in the current procurement process of off-patent medicines was too high according to the majority of workshop participants. As a consequence, it was initially reduced from 70% (specified by the current legislation) to 50%. Nevertheless, even the 50% initial cut-off point that participants voted for also appeared to be too tight and rigorous after validating the initial draft MCDA tool with test cases. According to the linear scoring function, this means that products with more than a 50% higher acquisition cost than the lowest price candidate should be assigned 0% of scores for price, still resulting in an emphasis on price that is too dominant. Therefore the price weight was reduced to 40%. Test cases also revealed the need to adjust the scoring function of the price, and so the cut-off point was increased to + 100%. This indicates the preference of workshop participants to choose more price premium when it brings other benefits expressed in other criteria. Overall, the application of test cases improved the understanding of workshop participants on how MCDA works and what their opinion on weights and scoring functions means in real world decisions.

### Comparison with other frameworks

The Chinese Beijing MCDA framework [17, 18] for off-patent tenders assigns a maximum of 100 points for non-price criteria (50 for product quality criterion and 50 for manufacturer site criterion, respectively) and an additional 30 points for price. Therefore, Beijing applies an even lower weight for price of 23.1% compared to the 40% weight recommended in Indonesia. However, as the test cases highlighted, the weight of price also highly depends on the scoring function for the price criterion. Similarly to the conclusion of the Indonesian workshop participants, the Beijing MCDA process also nominates multiple (namely two) winners.

### Proposed steps of policy implementation

Table 5 describes key steps to the implementation of a repeated use MCDA. The first 6 steps of the MCDA development and dissemination are already completed by running the policy workshop in Jakarta. However, further steps are necessary to have the final MCDA up and running in routine use in Indonesia for making OPP procurement decisions.

The action plan highlighted the necessity of standardizing the formal requirements of the submission dossier prepared by pharmaceutical companies. During the workshop, the test cases were provided to the participants in an easy-to-follow one-page performance matrix (Table 1), containing how the drug performed according to each criterion. Once the initial MCDA is being routinely used, a similar template should be considered as a cover page for pharmaceutical submission dossiers. This means that pharmaceutical companies should indicate scores based on the best available and submitted evidence for their pharmaceutical products and then the MCDA committee should validate initial scores after critical appraisal of the submitted evidence. At first, materials submitted by manufacturers should be checked by the secretariat of the procurement agency in order to prepare the initial performance matrix for each competing alternative. Some of the inputs submitted by the manufacturers, e.g. the supply track record of manufacturers or data on equivalence to the original product may be partially or fully validated by specific regulatory bodies, such as the Ministry of Health or the Indonesian drug regulatory agency. Then, based on the initial validation of scores, the cover page of the dossier containing an assessment on how the product performs according to the MCDA criteria shall be forwarded to a committee for final approval of the scores before making a recommendation.

### Uncertainty in the MCDA framework

In general, uncertainty in MCDA models can be related to model inputs and structural uncertainty [12]. As our MCDA framework is developed for repeated use, the uncertainty in input data should be managed at the critical appraisal phase by the secretariat of the procurement agency. Structural uncertainty can be related to disagreement on the criteria selection, weighting method or scoring functions. Although workshop participants represented all key institutions in Indonesia, the overall number of participants who participated in the voting was relatively low, which is a potential limitation of our study and represents room for structural uncertainty. Therefore, after a one-year pilot phase authors propose that the MCDA framework should be revisited based on the initial experience. Once the necessary adjustments on weights or scoring functions are made in a similar policy workshop, the final MCDA is ready to be officially introduced in the decision-making process. However, considering the periodic review of the MCDA tool (e.g. in every 3 years) may further reduce the structural uncertainty.

### Policy implications

Explicit and publicly available decision criteria can potentially further improve the transparency and accountability of the national procurement process in Indonesia. Additionally, the introduction of other criteria could create incentives for manufacturers to invest into additional data collection, improved supply chain reliability or better quality off-patent products which may ultimately result in more health gain for the society. The study confirmed the potential relevance of MCDA methodology and easy-to-use tools in the pharmaceutical policies of developing countries even for OPPs. Many of the learnings from this process in Indonesia can be applied to other emerging market countries as well.

## Conclusions

The study describes a prototype of how additional criteria can be added to improve the lowest price policy objective in public procurement of off-patent pharmaceuticals in a developing country. MCDA seems to be a useful tool for the drug procurement process (tender) in Indonesia. While according to the global trends, MCDA may facilitate several separate decision points in health care systems, the list of criteria, weights and scoring functions should be adjusted to the decision context. The Indonesian case highlighted that within the current tendering criteria, the pharmaceutical’s acquisition cost is too dominant, which may be corrected by 1) reducing the weight of price or 2) by increasing the cut-off point while aiming not to exclude good quality but higher-priced products from the opportunity to win a tender. Nevertheless, an equilibrium on price weight should be identified to maintain financial sustainability and political support to implement the new system. The explicit introduction of multiple non-price criteria suggests to pharmaceutical companies that their efforts to do more than what is required will be awarded.

## Abbreviations

BE: bioequivalence
BPOM: Badan Pengawas Obat dan Makanan
DJSN–JKN: Dewan Jaminan Sosial Nasional-Jaminan Kesehatan Nasional
GMP: Good Manufacturing Practice
IDR: Indonesian Rupiah
ISPOR: International Society For Pharmacoeconomics and Outcomes Research
JKN: Jaminan Kesehatan Nasional
MCDA: Multi-Criteria Decision Analysis
OPP: Off-Patent Pharmaceuticals
PE: pharmaceutical equivalence
PIC/S: Pharmaceutical Inspection Co-operation Scheme
PPK: Pejabat Pembuat Komitmen
SMART: Simple Multi-Attribute Rating Technique
UGM: Universitas Gadjah Mada
